# Supplementary material for: Deaf intermarriage has limited effect on the prevalence of recessive deafness and no effect on underlying allelic frequency
Source: PLoS One. 2020 Nov 4;15(11):e0241609. doi: 10.1371/journal.pone.0241609 (PMC7641374; doi:10.1371/journal.pone.0241609)
Supplement: Attachment — Submitted filename: Response to Reviewers.pdf [file pone.0241609.s001.pdf]

GALLAUDET UNIVERSITY

DEPARTMENT OF SCIENCE, TECHNOLOGY & MATHEMATICS
(202) 651-5385(V/TTY)
(202) 651-5521 (FAX)

KENDALL GREEN
800 FLORIDA AVE. NE
WASHINGTON, DC 20002-3695

July 30, 2020

Hela Azaiez, Ph.D.
Academic Editor, *PLOS ONE*

Dear Dr. Azaiez,

We are submitting a revision to manuscript PONE-D-20-07542,"Deaf intermarriage does not increase the prevalence of deafness alleles " for publication in PLOS ONE.

We have made significant revisions to the manuscript, in response to reviewer comments. Below, we describe these revisions. Reviewer comments are indented and noted in blue font.

We thank you and the reviewers for your comments, and believe that it's improved the manuscript. We are pleased with the results and hope that you will be, too.

Editor's Comments

> 1. Please ensure that your manuscript meets PLOS ONE's style requirements, including those for file naming.

Thank you. We went through these style requirements carefully and revised the manuscript to follow them.

> 2. Thank you for stating the following in the Acknowledgments Section of your manuscript:
>
> "We thank the NASA/D.C. Space Grant Consortium and the Beverly Taylor Sorensen Student Fellowship for their generous support of the student interns who worked on this project."
>
> We note that you have provided funding information that is not currently declared in your Funding Statement. However, funding information should not appear in the Acknowledgments section or other areas of your manuscript. We will only publish funding information present in the Funding Statement section of the online submission form.
>
> Please remove any funding-related text from the manuscript and let us know how you would like to update your Funding Statement. Currently, your Funding Statement reads as follows:

"The author(s) received no specific funding for this work."

Thank you for this feedback. We removed the funding text from the Acknowledgements section of the manuscript body and, instead, submitted the funding information through the submission system.

3. We note that you have stated that you will provide repository information for your data at acceptance. Should your manuscript be accepted for publication, we will hold it until you provide the relevant accession numbers or DOIs necessary to access your data. If you wish to make changes to your Data Availability statement, please describe these changes in your cover letter and we will update your Data Availability statement to reflect the information you provide.

In the first submission, we stated that the code was publicly available via a github repository. We modified this to say that both the code and the dataset are publicly available via the github repository:

> The source code and dataset created for this study are publicly available from https://github.com/derekbraun/homogamy.git so that anyone can replicate our experiments and build upon our work.

Reviewer #1's Comments

The introduction should be trimmed. There is a lot of superfluous information that isnt required.

Thank you for this feedback. We shortened the introduction from 98 lines to 79 lines by removing and condensing some of the information, mostly the historical background about AG Bell.

Lines 91-93: Three *GJB2* frameshift variants account for nearly all *GJB2* deafness, and are associated with specific ethnic groups: 35delG in European ancestry, 167delT in Ashkenazi Jewish ancestry, and 235delC in Asian ancestry [12- 14]. This is not true. The p.M34T and p.V37I are the most common contributors to *GJB2*-related hearing loss. The frameshifts the authors mention account for most of the severe to profound hearing loss cases associated with GJB2- related deafness. Please revise this.

Thank you for pointing this out. We revised this portion of the manuscript to make it clearer. We added language throughout the manuscript to remind readers that our study focuses on severe to profound deafness. The reason the study is limited to individuals with this degree of deafness is because that's the subset of American deaf individuals who learn American Sign Language and become pupils at residential schools for the deaf and marry other deaf individuals. Those with milder forms of deafness tend to remain with their hearing peers. p.M34T and p.V37I cause milder forms of deafness that usually do not result in students attending residential schools and/or learning ASL. We also revised the text to remind the readers

that these simulation results are not locus-specific and generalizable to autosomal recessive deafness other than *GJB2*.

> Please refer to the variants using the proper HGVS cDNA notation (ie 35delG to c.35delG).

We have changed all notation throughout the manuscript to HGVS notation. Thank you.

> Lines 97-98: It is unclear what the authors mean by acquired deafness. Just because the deafness is not congenital does not mean it does not have a genetic origin. Many forms of autosomal dominant hearing loss onset are post-lingual. Many genetic forms of mild-deafness are missed on many new born hearing screens and are picked up when a child starts school. These like the AD-deafness should not be considered acquired forms of deafness.

Thank you for pointing this out. We revised here, and throughout the manuscript that we were talking about deafness caused by causes other than the recessive allele that we are simulating. Biologically, this would mean deafness due to other complementary genes and/or morbidity.

> The authors need to use a more recent source that the Marazita ML et al 1993 study to estimate the frequency of acquired deafness. Over the past 27 year the understanding of the genetics of deafness and its epidemiology has drastically improved and this study might not be accurate.

Thank you, and we agree. The Marazita reference is particularly strong, so we kept that, but we are now also citing Morton (2006) and Yang (2019).

> Lines 142-146: Some of the terms the authors list cannot be used interchangeably. For example, inbreeding and assortative mating. These are not the same. One is based on genetic ancestry and the other is based on phenotype.

Thank you. We agree that these do not mean the same thing. After some thought, we decided to simplify the manuscript by using the term “assortative mating” throughout. We also clarified in the Background that while Fisher and Wright's mathematical model was developed for inbreeding, it is applicable to assortative mating. We hope this is more transparent/readable.

Roughly 6 generations (based on the authors calculation of 20year/generation) have passed since the Fay 1898 study. Has the rate of assortative mating among the deaf changed during this time?

The degree of assortative mating among deaf Americans has remained relatively stable over the past 200 years. There are three studies measuring the rate of assortative mating in deaf Americans: Fay (1898), Schein and Delk (1971), and Blanton *et al.* (2010). Weve added a paragraph with this detail. This is now mentioned both in the Background and Discussion sections.

How does the birth rate among hearing couples vs deaf-couples compare? Does this need to be considered?

The Blanton et al (2010) paper measured reproductive fitness based on the average number of children per family, which was normalized. There was a paragraph about this in the Discussion; we've added a similar paragraph to the Background with more detail.

Line 149: It is unclear where the 1.304% comes from. Please clarify. Please clarify the source of the allele frequencies.

Thank you for asking. We are sure that other readers will have the same question, so we added more detail to the manuscript. The 1.304% figure is taken directly from Nance and Kearsey (2004). We are using Nance and Kearsey's simulation parameters so that we can directly compare our computer simulation results against theirs. Nance and Kearsey did not identify in their essay a source for that particular figure; however, the frequency they used of 1.304% is at about the midpoint of the reported ranges of carrier frequencies in white Americans, ranging between 0.6% to 2.1%, so it is entirely reasonable (see for example Van Laer et al, 2001).

Do these results apply to only outbred populations?

In Fig 2 and Table 1 we show the results in the absence of assortative mating (i.e., when it is set to 0%). Neither the phenotypic or allelic frequencies change after 20 generations, as would be expected from Fisher's and Wright's model.

Line 266: delete .

Done. Thank you.

Line 267-278: The evidence for *GJB2-GJB6* digenetic inheritance is limited. It is now well established the large upstream deletions (some of which overlap *GJB6*) alter a cis-regulatory element of *GJB2*.

Line 273-278: The authors need to remove the hypothesis of a dominant digenetic *GJB2/GJB6* allele. The hypothesis does not make sense and the biologically basis for it is questionable.

Thank you for your feedback. We agree and have removed this paragraph altogether.

Line 285-293: Are the authors suggesting that haploinsufficiency of *GJB2* is protective against dysentery OR that complete loss of *GJB2* would be protective? It is unclear. If the latter, does that imply the rate of *GJB2*-related deafness would have increased during this time, given the survival advantage? It would be helpful for the authors to clarify this section.

Thank you for these questions. We've added some more detail to this paragraph, and clarified that this hypothesis remains fully untested. We also state that we don't know whether a heterozygote carrier might have partial protection against dysentery.

Line 297: Please add citations

Done. Thank you for pointing this out.

The rational of using the c.35delG, c.235delC and c.167delT variants only is confusing. It is also not clear why the authors use the frequency of these alleles only in the European population. The study might be more powerful if the authors used global population frequencies and use the frequencies for all loss of function alleles for GJB2.

We've clarified why there is so much emphasis on c.35delG, c.235delC and c.167delT throughout the paper, particularly in the Background and the Discussion. These three variants cause most of the severe to profound genetic deafness in the United States and in most world populations. The previous hypotheses and studies about assortative mating among deaf individuals were applied to the North American population. The frequency of various forms of genetic deafness are considerably different in each worldwide population so it would be difficult, and probably misleading, to try to use a single worldwide value.

Reviewer #2's Comments

Lines 166-167. What is U? is this Mann Whitney test statistics? Both the U value and p-value seems unusually high/low.

Lines 171-172. Similarly, why similar U values produce drastically different p-values?

Yes, this is the Mann-Whitney test (we added text for clarity). We ran the Mann-Whitney tests again and reconfirmed the results. We added further detail to the manuscript: $n = 5{,}000$ simulations (because at such large n, the Mann-Whitney test is very sensitive). We also calculated and now report the common language effect size, f, which is an unbiased estimator of the population value. We hope this makes the statistical analysis clearer.

Lines 167, 181, 217. Why the authors use 96% CI? This is very unconventional.

Thank you for pointing this out. We reexamined these values and agree that both our original choice of intervals, including how we used the intervals, was confusing, so we decided it was best to remove this and, instead, report n and the common language effect size, f.

Did the authors simulated any random mating cases and compare the intermarriage simulation with the random mating simulation to see if there is any difference?

Yes. The results for this comparison is seen in Fig 2 and Table 1.

Some references are not formatted correctly. See lines 163 and 196.

Thank you for your careful proofreading. We have fixed these two lines and also scanned the text to make sure we did not leave any unformatted citations.

With best wishes,

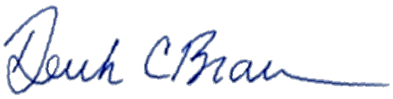

Derek C. Braun, Ph.D.
email: derek.braun@gallaudet.edu
