# Supplementary material for: Deaf intermarriage has limited effect on the prevalence of recessive deafness and no effect on underlying allelic frequency
Source: PLoS One. 2020 Nov 4;15(11):e0241609. doi: 10.1371/journal.pone.0241609 (PMC7641374; doi:10.1371/journal.pone.0241609)
Supplement: Attachment — Submitted filename: Response to Reviewers.pdf [file pone.0241609.s002.pdf]

GALLAUDET UNIVERSITY

DEPARTMENT OF SCIENCE, TECHNOLOGY & MATHEMATICS
(202) 651-5385(V/TTY)
(202) 651-5521 (FAX)

KENDALL GREEN
800 FLORIDA AVE. NE
WASHINGTON, DC 20002-3695

October 10, 2020

Hela Azaiez, Ph.D.
Academic Editor, *PLOS ONE*

Dear Dr. Azaiez,

We are submitting a revision to manuscript PONE-D-20-07542-R with the revised title, "Deaf intermarriage has limited effect on the prevalence of recessive deafness and no effect on underlying allelic frequency" for publication in PLOS ONE.

We made the revisions that Reviewer #1 asked for in their two comments. Below, Reviewer #1's comments are indented and noted in blue font, and below, we describe each revisions.

We thank Reviewer #1 for their comments. We are pleased with the results and hope that you will be, too.

Reviewer #1's Comments

> Line 283-286: This line is not correct. c.235delC is a founder mutation in the East Asians, c.167delT is a founder mutation in the Ashkenazi Jewish population and c.35delG is most common is Caucasians and white Europeans.

Agreed! We had inadvertently introduced this error while editing the paper and had failed to catch it during proofreading. Thank you for pointing this out.

> It is unclear how Fisher and Wright's mathematical model that was derived for inbreeding and assessing allele frequencies can be applied to phenotypes. With assertive mating, there is much variability at the genetic and allelic levels, however with inbreeding there is no new alleles being introduced. This needs to be further detailed.

While Fisher's paper was about inbreeding (but for the most part, applicable to assortative mating), Wright's paper was about assortative mating. Wright wrote a series of papers in 1921, and we had cited the wrong one. We have corrected this citation. Most importantly though, the theory and mathematics we are using are a re-derivation of Wright's and Fisher's work, that was done in 1968 by Crow and Felsenstein. Crow and

Felsenstein's paper is explicitly about assortative mating and actually uses the deaf community as a brief example.

We've clarified this in the manuscript and also added some more detail, that we hope makes the manuscript more understandable.

> There is a big difference between allele frequency and phenotypic frequency, specifically for a recessive disease. This is why countries with a high degree of inbreeding have a much higher rate of recessive diseases.

We understand and agree. The challenge, for us, in writing this manuscript was how to discuss both the prevalence of deafness and the underlying allelic frequencies - these two things are related but not the same thing. We can see how it would be easy to get our readers mixed up about which we are discussing. Your comments have caused us to go through the paper several more times, editing for clarity. We hope that these edits have made the paper clearer.

> A simpler way address what the authors want to is compare three simple scenarios:
>
> 1- The chance of having a deaf child if both parents are deaf due to the mutations in the same gene (ie 100% of their offspring will have hearing loss).
>
> 2- The chance of a deaf couple having a deaf child if both parents are deaf but different genes are responsible for their deafness.
>
> 3- The chance of a hearing couple having a deaf child.

Yes, this is true. We incorporated this simple demonstration in the additional detail that we added to our paper in the Background section.

Thank you, again, for your reading and your comments.

With best wishes,

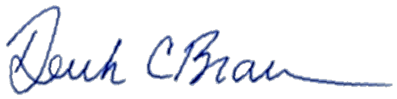

Derek C. Braun, Ph.D.
email: derek.braun@gallaudet.edu
