# Supplementary material for: Deaf intermarriage has limited effect on the prevalence of recessive deafness and no effect on underlying allelic frequency
Source: PLoS One. 2020 Nov 4;15(11):e0241609. doi: 10.1371/journal.pone.0241609 (PMC7641374; doi:10.1371/journal.pone.0241609)
Supplement: Attachment — Submitted filename: Response to Reviewers.pdf [file pone.0241609.s003.pdf]

GALLAUDET UNIVERSITY

DEPARTMENT OF SCIENCE, TECHNOLOGY & MATHEMATICS
(202) 651-5385(V/TTY)
(202) 651-5521 (FAX)

KENDALL GREEN
800 FLORIDA AVE. NE
WASHINGTON, DC 20002-3695

October 15, 2020

Hela Azaiez, Ph.D.
Academic Editor, *PLOS ONE*

Dear Dr. Azaiez,

We are submitting a revision to manuscript PONE-D-20-07542-R2, “Deaf intermarriage has limited effect on the prevalence of recessive deafness and no effect on underlying allelic frequency” for publication in PLOS ONE.

Thank you for your reading of the manuscript and your thoughtful comment. We made the revision that you asked for, as follows:

> I only have one final minor change to the last sentence of the conclusion " Therefore, Alexander Graham Bell’s fears of a “deaf variety of the human race” will not happen even if deaf intermarriage and assortative mating continue its present course." Although I understand what you are referring to, I believe replacing the word "fears" with concept or notion would be better perceived by the Deaf community and readers. We invite you to submit a revised version of the manuscript with the change requested above.

Dr. Greenwald, one of the co-authors, is a historian who studies Bell’s writing and that of other eugenicists of his era. We settled on the word “concerns” because A.G. Bell’s polemic about deaf intermarriage was consistently strong and had a lasting impact. We hope this is acceptable.

With best wishes,

Derek C. Braun

Derek C. Braun, Ph.D.
email: derek.braun@gallaudet.edu
